# Risk of poisoning in children and adolescents with ADHD: a systematic review and meta-analysis

**DOI:** 10.1038/s41598-018-25893-9

**Published:** 2018-05-15

**Authors:** Maite Ruiz-Goikoetxea, Samuele Cortese, Sara Magallón, Maite Aznárez-Sanado, Noelia Álvarez Zallo, Elkin O. Luis, Pilar de Castro-Manglano, Cesar Soutullo, Gonzalo Arrondo

**Affiliations:** 1 Servicio de Urgencias Extrahospitalarias, Servicio Navarro de Salud-Osasunbidea, Pamplona, Spain; 2 Center for Innovation in Mental Health, University of Southampton, Academic Unit of Psychology, Southampton, UK; 3 Faculty of Medicine, Clinical and Experimental Sciences (CNS and Psychiatry), University of Southampton, Southampton, UK; 4 Department of Child and Adolescent Psychiatry, NYU Langone Medical Center, New York, NY USA; 5 Solent NHS Trust, Southampton, UK; 6 Division of Psychiatry and Applied Psychology, School of Medicine, University of Nottingham, Nottingham, UK; 7 Facultad de Educación y Psicología, Universidad de Navarra, Pamplona, Spain; 8 Unidad de Psiquiatría Infantil y Adolescente, Departamento de Psiquiatría y Psicología Médica, Clínica Universidad de Navarra, Pamplona y Madrid, Spain; 9 Instituto Cultura y Sociedad (ICS), Grupo Mente-Cerebro, Universidad de Navarra, Pamplona, Spain

## Abstract

Poisoning, a subtype of physical injury, is an important hazard in children and youth. Individuals with ADHD may be at higher risk of poisoning. Here, we conducted a systematic review and meta-analysis to quantify this risk. Furthermore, since physical injuries, likely share causal mechanisms with those of poisoning, we compared the relative risk of poisoning and injuries pooling studies reporting both. As per our pre-registered protocol (PROSPERO ID CRD42017079911), we searched 114 databases through November 2017. From a pool of 826 potentially relevant references, screened independently by two researchers, nine studies (84,756 individuals with and 1,398,946 without the disorder) were retained. We pooled hazard and odds ratios using Robust Variance Estimation, a meta-analytic method aimed to deal with non-independence of outcomes. We found that ADHD is associated with a significantly higher risk of poisoning (Relative Risk = 3.14, 95% Confidence Interval = 2.23 to 4.42). Results also indicated that the relative risk of poisoning is significantly higher than that of physical injuries when comparing individuals with and without ADHD (Beta coefficient = 0.686, 95% Confidence Interval = 0.166 to 1.206). These findings should inform clinical guidelines and public health programs aimed to reduce physical risks in children/adolescents with ADHD.

## Introduction

Poisoning is defined by the World Health Organization as “an injury that results from being exposed to an exogenous substance that causes cellular injury or death”^1^. Poisons can be inhaled, ingested, injected or absorbed. On a global scale, poisoning is estimated to cause 350,000 deaths every year, of which 45,000 refer to individuals under the age of twenty^1^, and, more generally, it leads to higher mortality and morbidity rates in this age group^2–6^.

Risk factors for poisonings include age and sex, among others. Being male is related to a higher poisoning risk across all age groups^1,5^. The relationship between age and risk of poisoning has a bimodal distribution with two peaks of highest risk between the ages of 1 and 4, as well as between 13 and 18 years of age^1,5^. Age is also associated with a change in the mechanism of poisoning: whereas most poisonings before the age of fourteen are unintentional, the proportion of intentional poisonings increases dramatically from that age onward^1,7^. The majority of suicide intents in adolescents consist of intentional intoxications^1,6^, accounting for one third of total poisonings in that age range^5^.

Attention-deficit/hyperactivity disorder (ADHD) has a world-wide estimated prevalence of around 5%^8^, which makes it the most frequent neurodevelopmental disorder in children and adolescents. It is characterized by inattentive and/or hyperactive- impulsive symptoms that have a negative impact on social^9^, academic^10^, and health domains^11–13^, and reduce the quality of life^14^. ADHD is approximately four times more common in boys than in girls. Pharmacological treatment, including psychostimulants (methylphenidate and amphetamines) and non-psychostimulants (e.g., atomoxetine, guanfacine), is an important component of the multimodal treatment of ADHD^15^. In addition, a high percentage of patients have comorbid disorders hence increasing the probability of patients being poly-medicated^16^.

A recent meta-analysis by our group has demonstrated that the risk of physical injuries is significantly higher in children and adolescents with ADHD compared to the typically developing population. Additionally, this risk is significantly reduced by the use of ADHD medications^17^. Therefore, a plausible hypothesis is that ADHD symptoms (inattention, hyperactivity and impulsivity) could lead to a similar increase in the risk of poisoning. Impulsivity might be an important factor, especially considering that it is significantly associated with suicide attempts, as shown in a recent meta-analysis^18^. Indeed, a recent systematic review on the relationship between ADHD and suicide concluded that there is a positive association between ADHD and suicidality in both sexes and in all age groups that was likely mediated by the presence of comorbid disorders^19^. Furthermore, individuals with ADHD frequently have more access to potentially harmful medications that many of them take either for the disorder or for its comorbidities.

Whereas a higher rate of poisoning in children and adolescents with ADHD in comparison with their typically developing peers has been reported in individual studies, the magnitude of the association is unclear^20,21^. Therefore, a meta-analysis quantifying the risk of poisoning in children/adolescents with ADHD is timely. Of note, the previous meta-analysis on the risk of physical injuries excluded studies that specifically analyzed the risk of poisoning^17^. To fill this gap and complement the previous meta-analysis, we conducted the present meta-analysis aimed to quantifying the pooled risk of poisoning in children/adolescents with ADHD compared to non-ADHD controls. A secondary aim was to compare the magnitude of the risk of unintentional physical injuries and poisoning from studies that reported both. We hypothesized that children and adolescents meeting criteria of ADHD would have significantly higher rates of poisoning compared to those without ADHD, and this increased probability of poisoning would be greater than that of physical injuries.

## Results

**Figure 1:**
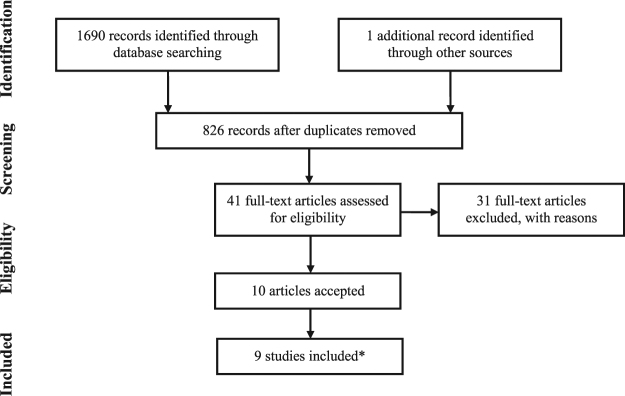
PRISMA flow diagram of record identification and study selection. *Four additional articles only reporting physical injuries for two of the included studies were also found.

Searches carried out in 114 databases (including three major bibliographic databases plus 111 additional resources from a database aggregator) in November 2017 led to 826 articles, whereas nine studies were included in the final stage of the systematic review and meta-analysis^20–29^. Articles that were considered possible candidates for inclusion during the first screening stage but were later deemed ineligible when the full text was assessed are listed in Table S1, with reasons for exclusion (see Supplementary material). Multiple reports derived from the Taiwan Longitudinal Health Insurance Database (LHID) were treated as the same study^20,25,30–32^, similarly to the Clinical Practice Research Datalink and Hospital Episode Statistics (CPRD-HES) from the United Kingdom^23,33^. The full process of article search and selection is shown in the PRISMA flow diagram in Fig. 1. Details from the included studies are reported in Table 1 (overall description) and Table 2 (identification of poisoning). Outcome-level data extracted from each article on the risk of poisoning can be found in Table 3 (16 outcomes in total).

**Table 1:** Description of studies included in the meta-analysis.

Name	Country	Sample	Type of study	N non-ADHD	N ADHD	ADHD diagnosis	% Male ADHD	% Male Control	Medicated %	Duration	Age range	Risk Measure
Brehaut^22^	Canada	BCLHD	Registry	1010067	16806	Administrative coding (medication)	81,6	50,9	100	>1	0–19	OR
CPRD-HES^23,33^	UK	CPRD-HES	Registry	291909^c^	15742^c^	Administrative coding	84,6	50,7	44	>1	3–17	HR
Hariharan^21^	US		Registry (Case-control)	87	16	Administrative coding (medication and ICD-9)	NR	NR	45	NA	5–9	OR
Hurtig^24^	Finland	NFBC	Prospective cohort	5639^a^	288^d^	Clinical/Scales with threshold	66.1^a^	48.5^a^	0	>1	0–15	HR
LHID^20,25,30–32^	Taiwan	LHID	Registry	36850^ac^	3685^ac^	Administrative coding	79.0^ac^	79.0^ac^	74.3^ac^	>1	3–18	OR and HR
Lindemann^26^	Germany	GEPARD	Regystry	37650	37650	Administrative coding (medication and ICD-10)	NR	NR	NR	>1	3–17	HR
Rowe^27^	UK	BCAMHS:99	Population-based survey	10073^b^	365^b^	DSM-IV	NR	NR	NR	>1	5–15	OR
Silva^28^	Australia	MNS	Registry	5363	8896	Administrative coding (ICD-9 and ICD-10)	78.3	78	100	>1	0–4	OR
Swensen^29^	US		Registry	1308	1308	Administrative coding (Other ICD)	73.2	73.2	NR	<1	Any (17% over 18)	OR

Country: country where data were collected; Sample: abbreviated name of the sample originating the data; % medicated: percentage of medicated individuals with ADHD, duration is duration of follow-up time for occurrence of injuries and is reported as <1 (less or equal to a year) or >1 (more than a year); Age range at injury; NR: not reported; NA: not applicable.

^a^Obtained from biggest outcome.

^b^Estimated from total (3.5% of total for individuals with ADHD and 96.5 for individuals without ADHD)^69^.

^c^Number obtained from article(s) reporting poisoning.

^d^Averaged between outcomes.

**Table 2:** Identification of poisoning cases.

Name	Method for diagnosis	Classification system(s): codes	Types of poisoning
Brehaut (2003)^22^	Registry	ICD-9: 960–989	Medicinal and non-medicinal
CPRD-HES: Prasad (2016)^23^	Registry	ICD-10 and OPCS4	Medicinal and non-medicinal
Hariharan (2008)^21^	Registry	ICD-9	Medicinal (self-taken, not inhaled or by contact).
Hurtig (2016)^24^	Registry	ICD-8, ICD-9 and ICD-10	Medicinal and non-medicinal
LHID: Tai (2013)^20^	Registry	ICD-9: 960–989	Medicinal and non-medicinal^70^
LHID: Chou (2014)^25^	Registry	ICD-9: 960–979 and E930–949	Medicinal (deliberate)
Lindemann (2017)^26^	Registry	ICD-10 T36–75, T96–97	System-wide injuries^71^
Rowe (2004)^27^	Self-report	NA	Medicinal and non-medicinal
Silva (2014)^28^	Registry	ICD-9 and ICD-10: T36-T65	Medicinal and non-medicinal
Swensen (2004)^29^	Registry	ICD-9: 960–989	Medicinal and non-medicinal

Registry indicates that a retrospective registry was used to identify poisoning cases. OPCS4: Office of Population Censuses and Surveys (OPCS-4) version 4. NA: not applicable.

**Table 3:** Outcome-level details of all the outcomes extracted from the studies included in the risk of poisoning analyses.

First author (year)	Measure	Description of outcome	N non-ADHD	N ADHD	Number of non-ADHD poisoned	Number of ADHD poisoned	Prevalence (per 1000) of poisoning in non-ADHD	Prevalence (per 1000) of poisoning in ADHD	Relative Risk	LBCI	UBCI	Main Analysis
Brehaut (2003)^22^	OR	Adjusted	1010067	16806	3882	184	3.8	10.9	2.67	2.27	3.14	Yes
CPRD-HES: Prasad (2016)^23^	HR	Adjusted	291909	15742	2033	463	7.0	29.4	3.99	3.58	4.44	Yes
Hariharan (2018)^21^	OR	Unadjusted	87	16	20	11	NR	NR	7.98	2.64	24.13	Yes
Hurtig (2016)^24^	HR	Rating scale, injury between 0 and 6 years. Adjusted.	5236	875	44	14	8.4	16.0	1.51	0.76	3.01	Yes
Hurtig (2016)^24^	HR	Rating scale, injury between 7 and 15 years. Adjusted	5639	472	27	9	4.8	19.1	3.42	1.46	8.02	Yes
Hurtig (2016)^24^	HR	Clinical criteria, injury between 7 and 15 years. Adjusted	352	105	3	2	8.5	19.0	6.29	0.8	49.35	Yes
LHID: Tai (2013)^20^	OR	Unadjusted	7860	1965	98	30	12.5	15.3	1.23	0.81	1.85	No
LHID: Chou^2^ (2014)^5^	HR	Unadjusted	36850	3685	29	13	0.8	3.5	4.51	2.35	8.68	No
LHID: Chou (2014)^25^	HR	Adjusted.	36850	3685	29	13	0.8	3.5	4.65	2.41	8.94	Yes
LHID: Chou (2014)^25^	OR	Unadjusted	36850	3685	29	13	0.8	3.5	4.50	2.33	8.65	No
LHID: Chou (2014)^25^	HR	0 to12 years. Adjusted.	NR	NR	29	13	NR	NR	2.42	0.99	5.89	No
LHID: Chou (2014)^25^	HR	12 to 18 years. Adjusted.	NR	NR	29	13	NR	NR	17.86	5.23	61.02	No
Lindemann (2017)^26^	HR	Adjusted	37650	37650	NR	NR	NR	NR	3.47	2.14	5.64	Yes
Rowe (2004)^27^	OR	Psychiatric model. Adjusted	NR	NR	NR	NR	NR	NR	1.2	0.5	2.6	Yes
Silva (2014)^28^	OR	Adjusted	8896	5363	332	322	37.3	60.0	2.24	1.91	2.65	Yes
Swensen (2004)^29^	OR	Unadjusted	1308	1308	5	22	3.8	16.8	4.46	1.68	11.81	Yes

N: number of individuals in each group; OR: odds ratio between children and adolescents with ADHD and without ADHD; HR: hazard ratio between children and adolescents with ADHD and without ADHD; LBCI: lower bound of the 95% confident interval; UBCI: upper bound of the 95% confident interval; Main analysis: it indicates if the outcome has been included in the main analysis (most controlled and general outcome) and in the main sensitivity analyses. NR: not reported.

The origin of the studies was varied, comprising North-America, Europe, Asia and Australia. All studies but one^21^ were based on large epidemiological databases. More specifically, there were two regional^22,28^ and three national databases^20,23,26^, a nationally representative survey^27^, a population-based prospective cohort^24^, a study using administrative claims from a self-insured company^29^, and a case-control study using a hospital-based registry^21^. Therefore, all studies but two^21,27^ analyzed administrative databases not specifically designed for research purposes at their inception^34^. A strength of the included studies is that they tended to have large sample sizes (between 87 and 1,010,067; median 10,073; for the controls and between 16 and 37,650; median 3,685; for ADHD). The systematic review and meta-analysis pooled data from a total sample of 84,756 and 1,398,946 children and adolescents with and without ADHD respectively.

Overall, poisoning cases were uncommon. The median number per study of poisoned individuals that suffered from ADHD was 14 (range 2–184), whereas the median number per study of poisoned individuals who did not suffer from ADHD was 29 (range 3–3,882). Prevalence (per 1000) ranged between 3.5 and 60 (median 16) in children and adolescents with ADHD and between 0.8 and 37.3 (median 4.8) in children and adolescents without ADHD.

The ranges of ages of poisoning were large in most cases. An exception was a study in which ADHD was diagnosed in school-age children but retrospectively considered the risk of poisoning during pre-school^28^. This is a probable cause for the much higher risk of poisoning in both the group with and without ADHD in this study. The retrospective nature of studies and the use of administrative databases were also related to the type of strategies used to identify cases with ADHD and to define poisoning. In the majority of studies, ADHD diagnosis was defined based on ICD codes at visit discharges^20,23,25,28,29^, by taking medications for ADHD^22^, or based on the combinations of the two. However, two studies^24,27^ used scales of symptoms and DSM criteria. Similarly, diagnoses of poisoning were defined based on ICD codes. Whereas most studies included poisoning from medicinal and non-medicinal origins, two studies^21,25^ only included poisoning from medicinal drugs. Specifically, one^25^ analyzed poisoning cases that were intentional in nature, which led to a much smaller prevalence of poisoning in in adhd and controls.

**Figure 2:**
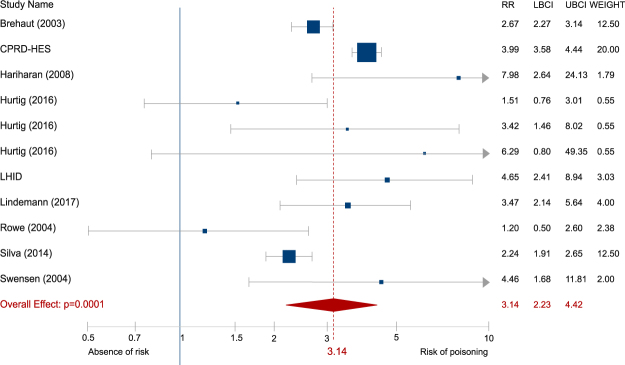
Pooled effect size estimating the association between ADHD and poisoning. Hazard and odds ratios were combined. The area of each square is proportional to the weight that the individual study contributed to the meta-analysis. Weights are from a ramdom-effects model using RVE. The diamond indicates the overall weighted mean effect across all studies. Study name is the first author and year except when several articles come from the same database. RR: relative risk, UBCI: upper bound of the 95% confidence interval, LBCI: lower bound of the 95% confidence interval

**Figure 3:**
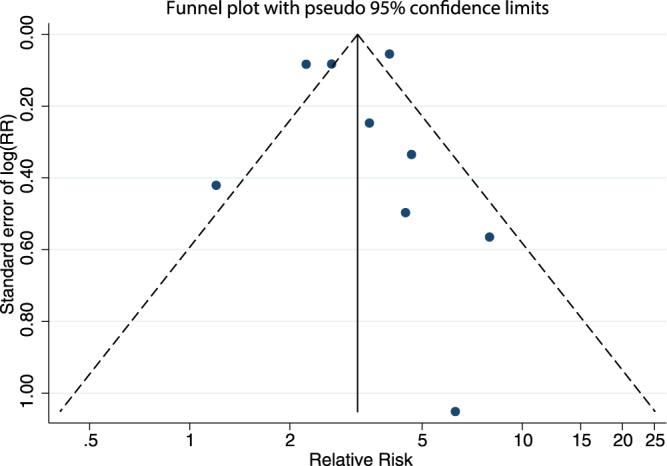
Funnel plot of the studies included in the risk of poisoning meta-analysis.

The main analysis, showing the relative risk (RR) of poisoning between adolescents with and without ADHD, included eleven outcomes derived from nine studies. Variation among effect sizes was important as they ranged between 1.2 and 7.98 (median 3.47). The overlap between confidence intervals (CIs) was small. The lower bound of the CI ranged between 0.5 and 3.58 (median 1.91) and the upper bound between 2.6 and 49.35 (median 5.64). All analyses were carried out using Robust Variance Estimation (RVE) to take into account dependence between outcomes. Individuals with ADHD had a significantly increased risk of poisoning compared to individuals without the disorder (RR = 3.14, 95% CI- = 2.23 to 4.42) as shown in the forest plot in Fig. 2. Heterogeneity of studies, as measured by Cochran’s Q test and I^2^ index^35^, was high (χ^2^ = 49.42, df = 8, p < 0.001, I^2^ = 83.8%). Risk of small sample bias was not significant according to Begg’s adjusted rank correlation and Egger’s test (Egger t = −0.07, p = 0.949; Begg Z = 0.52, p = 0.602, see also the funnel plot in Fig. 3).

In general, results were relatively robust to sensitivity analyses. When only studies using hazard ratios as effect measure were included, the resulting average RR was 3.91 (95% CI = 3.41 to 4.5, I^2^ = 0%). The average RR was 2.59 (95% CI = 1.81 to 3.71, I^2^ = 64.4%) when only odds ratios were taken into account. Similarly, the pooled RR changed to 3.01 (95% CI = 2.01 to 4.50, I^2^ = 87%) when only statistically adjusted RRs were entered in the meta-analysis, and increased to 5.62 (95% CI = 2.51 to 12.61, I^2^ = 0%) when uncontrolled effect sizes were used. Since only two studies did not combine poisoning and intoxication cases (defined using the ICD codes), we could not carry out a sensitivity analysis including only studies that focused on a strict definition of poisoning. It must be noted that in the case of crude effect sizes and hazard ratios only three and four studies were respectively included in the analyses and, therefore, confidence intervals with RVE are unreliable^36^. Changing the p parameter within RVE, a value that accounts for the correlation between outcomes within studies, did not change the previously stated estimation of effects.

Regarding the risk of bias, ratings on the Newcastle-Ottawa Scale (NOS) tended to be high (range 3 to 6, median 5, out of 7 possible stars as 2 items were deemed inadequate for our study). The items of the scale used in the present meta-analysis, subscores and total score for each study can be found in Table 4. A meta-regression including the NOS^37^ scores as a regressor showed no significant effects (Beta Coefficient-B- = −0.060, 95% CI = −1.087 to 0.967, p = 0.843).

**Table 4:** Newcastle Ottawa Scale scores.

Name	NOS version	Selection (up to 3 stars)^a^	Comparability(up to 2 stars)	Outcome/Exposure (up to 2 stars)^a^	NOS total (up to 7 stars)
Brehaut^22^	Cohort	2	1	1	4
CPRD-HES: Prasad^23^	Cohort	3	1	1	5
Hariharan^21^	Case-control	1	1	1	3
Hurtig^24^	Cohort	2.5*	1	1	4.5
LHID^20,25^	Cohort	3	1	1	5
Lindemann^26^	Cohort	3	1	1	5
Rowe^27^	Cohort	3	2	0	5
Silva^28^	Cohort	3	1	1	5
Swensen^29^	Cohort	3	2	1	6

Number of stars for each subsection of the Newcastle-Ottawa Scale (NOS) and the total score.

^a^An item from the original scale was not relevant for our meta-analysis (see Supplementary Material, S3 Methods).

*Averaged between outcomes.

Similarly, the sub-group analyses carried out in order to assess the effect of age were not statistically significant: results of the between-studies comparison of outcomes in children under ten years old against outcomes in which age was not specified were B = 0.299, 95% CI = −0.404 to 1.000 p = 0.279; when outcomes from participants with unspecified age were compared to outcomes obtained from participants over 10 years, results were not significant (B = −0.417, 95% CI = −0.737 to 1.571 p = 0.185); finally, when outcomes from individuals under and above 10 were compared, results were not significant either (B = −0.042, 95% CI = −2.080 to 1.996 p = 0.937).

**Figure 4:**
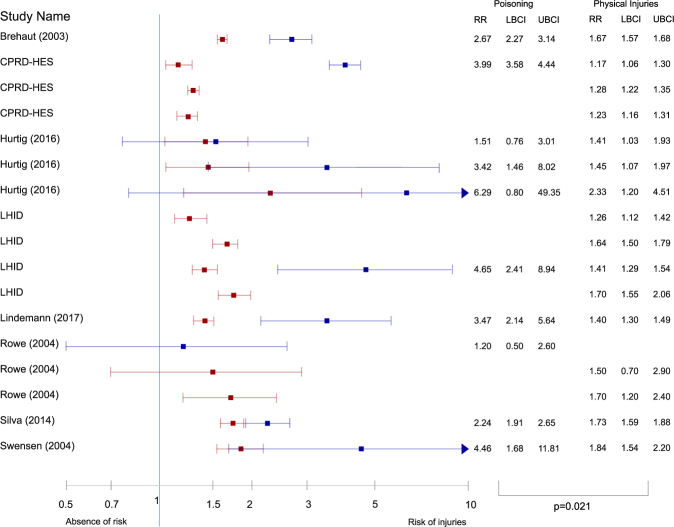
Comparison between the relative risk of poisoning and that of physical injuries in ADHD. Hazard and odds ratios from studies which reported both physical injuries and poisoning outcomes were combined. Poisoning relative risks are shown in blue and relative risks of physical injuries in red. Study name is the first author and year, except when several articles come from the same database. RR: relative risk, UBCI: upper bound of the 95% confidence interval, LBCI: lower bound of the 95% confidence interval. p value is obtained from a within-study analysis and indicates that the relative risk of poisoning is greater than that of physical injuries in children and adolescents with ADHD compared to their peers.

An important question of our systematic review and meta-analysis was whether the relative risk of poisoning was statistically different from the relative risk of suffering physical injuries in general. In order to answer this question, we extracted effect sizes reporting the relative risk of unintentional injuries from studies that reported both. Eight studies reported outcomes for both poisoning and physical injuries and one study^21^ reported only poisoning outcomes. The outcomes related to physical injuries (10 in total) are summarized in Table S3 (see supplementary material). The pooled RR of physical injuries was 1.54 (95% CI = 1.33 to 1.78). Heterogeneity of studies was significant (χ^2^ = 64.72, df = 7, p < 0.001, I^2^ = 89.2%). Risk of small sample bias was not significant (Egger t = −0.27, p = 0.798; Begg Z = 0.37, p = 0.711). The relative risk of physical injuries was significantly smaller than the one in the case of poisoning (B = 0.686, 95% CI = 0.166 to 1.206, p = 0.021). A forest plot comparing the two combinations of outcomes, with effect measures from studies which reported both physical injuries and poisoning outcomes, is shown in Fig. 4.

The confidence intervals of the pooled estimates of physical injuries reported in the present meta-analysis (ES = 1.54, 95% CI = 1.33 to 1.78, derived from the combination of studies that also reported estimates of poisoning), and those in our previous article (ES = 1.53, 95% CI = 1.40 to 1.67, derived from any study reporting injuries), did not overlap with the confidence intervals for the overall effect of poisoning (ES = 3.14, 95% CI = 2.23 to 4.42)^17^. The same occurred when the analysis was limited to studies reporting hazard ratios, as the confidence intervals reported in our previous article for the pooled estimates of physical injuries (HR = 1.39, 95% CI = 1.06 to 1.83) did not overlap with the confidence intervals for the overall effect of poisoning (HR = 3.91, 95% CI = 3.41 to 4.40)^17^. This is further evidence to support that the relative risk of poisoning is significantly greater than the relative risk of suffering physical injuries in children and adolescents with ADHD.

## Discussion

Poisoning is an important cause of morbidity among children and adolescents worldwide. Children with ADHD may represent a particularly vulnerable group, but so far, no pooled estimates of this risk were available. The present meta-analysis has concluded that there is a significantly higher risk of poisoning in children and adolescents with ADHD compared with their non-ADHD peers, with an estimated relative risk of 3.14 (95% CI = 2.23 to 4.42). Taking into account the prevalence of ADHD, the disorder could be a major factor contributing to the overall number of pediatric poisonings. Results derive from the combination of nine large population-based studies with a combined sample close to 1.4 million children and adolescents from the general population and 85k individuals with ADHD. The confidence interval of the estimate is quite narrow (95% CI = 2.23 to 4.42) because of the large sample size.

This increased risk is consistent with previous research, showing a significantly higher risk of physical injuries in individuals with ADHD^38^. Specific features of the disorder such as impulsivity and inattention are likely a major risk factor for poisoning. Our work provides solid meta-analytic evidence further highlighting that ADHD is a disorder with consequences that are not limited to the behavioral or educational domains. It has an impact on different health aspects^39^ and hence, leads to a reduction in the overall quality of life of the patients and their families^14^.

Results were generally robust to different sensitivity analyses. Furthermore, there was no statistical indication of small-sample bias, including publication bias. However, these analyses were limited by the final number of outcomes and studies in our meta-analysis, so that our results could partly reflect a lack of statistical power. A possible qualitative interpretation of the funnel plot related to our analyses is that studies with greater standard errors (typically smaller studies) were more likely published if they showed an increased risk of poisoning in children/adolescents with ADHD. If real, hidden reporting bias could be leading to an overestimation of the overall effect. However, a line of evidence makes this overestimation unlikely. In most published studies, relative risk of poisoning was only a secondary outcome among several other subgroups of injuries, whereas the central finding of these studies was an overall increased risk of injuries. In such case, publication bias due to results of only one of many secondary outcomes is unlikely. Moreover, within study comparisons are robust to publication biases. If there was an overall publication bias towards a higher risk of injuries in ADHD (and not only of poisoning), our within-study analytic strategy demonstrating that the relative risk of poisoning is greater than the relative risk of physical injuries in general would not be affected.

As already mentioned, whereas the pooled sample in our meta-analysis was very big, the number of studies was limited. This can be explained by the fact that poisoning cases are a rare event and, therefore, very large databases are typically needed to be able to carry out epidemiological studies on this outcome. These large epidemiological studies are difficult to conduct: on the one hand, prospective cohorts are expensive and limited; on the other hand, administrative databases (that is, databases for which the main aim and design was not initially research)^34^ are also limited in number and in many cases will not include measures of interest for the specific research question. Both issues, number of studies and measures reported in them, limited our analyses. We were not able to carry out possibly informative regression analyses, such as evaluating a relationship between the risk of poisoning and ADHD symptoms, or controlling for comorbidity. Similarly, the statistical power of our regression analyses was also very low. Indeed, as it is always the case in frequentist statistics, a lack of significance only indicates that there is not enough evidence for an effect, which does not necessary involve that there is not such an effect. Given that our statistical power was limited due to the characteristics of existing research, our results leave an open door for future studies evaluating such effects.

It is also noteworthy that despite our strict inclusion criteria, heterogeneity among studies was significant. Heterogeneity was dealt through the use of random-effects models that assume that the true effect size might differ from study to study, and that studies included in the analysis are a random sample of all possible studies that meet the inclusion criteria. Additionally, we investigated the origins of heterogeneity through meta-regression. The most influential factor driving heterogeneity was the type of outcome measure (Hazard or odds ratios), which influenced not only the pooled effect size (RR = 3.91, 95% CI = 3.41 to 4.5 for HR; RR = 2.59, 95% CI = 1.81 to 3.71 for OR) but also the I^2^ statistic (64.4% when only OR were included and 0% when only HR were included). However, this difference between results cannot be explained by the different outcome measure per se but from other study characteristics that co-occur with the selection of the outcome measure. Taking into account the fact that studies reporting HR tended to be larger, more representative of the population, had better statistical control of possible confounders, and the heterogeneity of their results was smaller, the estimation obtained when only including HR studies might better reflect the true relative risk of poisoning in children and adolescents with ADHD. It must be noted, however, that only four studies were included in the case of HRs and hence the confidence intervals calculated through RVE could be wider than expected^36^.

An additional major finding of the meta-analysis is that the relative risk of poisoning in individuals with ADHD compared to individuals without it was statistically higher than the overall relative risk of physical injuries. Eight studies reported both injuries in general and poisoning cases, hence permitting a within-study evaluation of the effect of type of injury. Of note, the risk of injuries from the combination of the eight studies was 1.54 (95% CI = 1.33 to1.78), closely matching the results from our previous meta-analysis on the risk of physical injuries, in which the mean RR was 1.53 (95% CI = 1.40 to 1.67). Several factors could be accounting for this increased relative risk. Accidental overdoses due to a difference between the taken and prescribed doses are common in pediatric populations^40^, and they increase with an easier access to pharmacological drugs. Indeed, access to medications has been reported as a risk factor for unintentional poisoning^41,42^. Children and adolescents with ADHD have more access to medications than developmentally normal individuals do. Nearly 60% of ADHD diagnosed children receive pharmacological treatment with stimulants and other drugs^43,44^, and in many cases a single individual will be prescribed several formulations of the same medication^45^. In addition, many individuals will receive additional medications for comorbid disorders^16^, such as oppositional defiant disorder, conduct disorder, anxiety, coordination problems, depression, tic disorders and Tourette syndrome. Hence, the poly-pharmacy status in many ADHD patients could increase the likelihood of an accidental poisoning. Moreover, comorbid mental disorders might make children and adolescents with ADHD even more prone to an accidental poisoning. Data on comorbidity was not reported in most of the studies included in the present meta-analysis, so that the impact of comorbidity could not be elucidated. Further research should clarify whether medicated individuals are at a greater risk of poisoning, if comorbidities increase the risk of intoxications, and to which extent these effects can be disentangled. The role of ADHD medication is even more complex. ADHD pharmacological treatment has already been shown to reduce the risk of suffering an unintentional injury^17^, and conversely, it reduces the risk of driving accidents in adults^46,47^. Drugs used to treat ADHD could have a similar effect on the risk of poising: as they improve attention and impulsivity, they could lead to a reduction in the risk of poisoning. However, our systematic review was not able to find any studies on the effect of medication on poisoning risk. Therefore, the relationship between medication effects and risk of poisoning in ADHD deserves further clarification.

In terms of age effects, poisoning incidence has two peaks across the child life span. The first peak occurs in the first years of life and the second is around the beginning of adolescence, changing also the causal factors of poisoning^1,5^. As the child grows, there is an increase of intentional poisonings, although the total percentage of intentional poisonings remains lower than the percentage of unintentional cases^1,5^. For the second age group, recreational drug usage and suicide attempts are important causes of poisoning. Regarding the specific case of individuals with ADHD, adolescents with the disorder use more frequently drugs recreationally^48^, including their own medications^49^. For example, a study carried out among adolescents and young adults with the disorder reported that 14.3% of the participants in the study had once abused of their prescribed pharmacological treatment^50^. Furthermore, evidence tends to support the fact that individuals with ADHD have a higher risk of suicide and suicide attempts^19^. In summary, ADHD adolescents could be especially prone to intentional (suicide attempts) or semi-intentional (recreational drug use) cases of poisoning, compared to younger children with the disorder and also the general population, and this could be driving in part the higher risk of poisonings compared to physical injuries. However, a direct test of this hypothesis was not possible in our meta-analysis since included studies did not differentiate between intentional and unintentional poisonings. Whereas future studies should try to address this issue, it must be noted that this differentiation is likely impossible when using administrative databases. We sought for indirect support for the role of intentional poisoning through a meta-regression including age as a covariate, but the results of this meta-regression analysis were not significant. Therefore, the role of age as a mediator in this issue still remains unknown.

The results of our systematic review/meta-analysis should be considered in the light of its strengths and limitations. As for the strengths, we pre-registered the protocol in a publicly available repository (PROSPERO), reducing the risk of reporting bias. Furthermore, we endeavored to perform a comprehensive and systematic search in several databases, with no restrictions in terms of language, date, or document type. Additionally, we used a state-of-the-art tool, the Newcastle-Ottawa scale, to assess the quality of the retained studies. Furthermore, the included studies typically used big longitudinal or administrative cohorts or national surveys, which provide adequate statistical power to estimate the overall incidence of an infrequent type of event, such as is poisoning. There are also a number of limitations that should be taken into account, which are mostly related to the individual studies that we included rather than to methodological issues with our systematic review/meta-analysis. First, intentionality of poisoning was not controlled in the included studies. Second, we could not find sufficient data to evaluate the effects of age, medication status or comorbidities on the risk of poisoning in ADHD. Since these major confounders were not controlled for in our analyses, the increased relative risk cannot be directly ascribed to ADHD. Although our results support the conclusion that individuals with ADHD in the real world suffer more poisoning events than those without it, we cannot know what factor or factors are at the origin of this relationship and in this regard, any causative explanation derived from them should be taken with caution.

Our findings have important implication from a public health standpoint. Poisoning remains a leading cause of preventable injuries in childhood and adolescence^1^, whose treatment involves a huge cost of economic and human resources^51,52^. The present meta-analysis has shown that children and adolescents with ADHD are a population with an increased risk of poisoning. Specific preventive measures in this population could help to minimize this risk or the detrimental consequences of poisoning. Health providers should ensure a correct understanding of treatment dosages and frequency intakes, as well as alarm signs regarding side effects or poisoning and how should parents and or patients act in a case of possible poisoning. They should also emphasize the hazard of having dangerous household products out of the reach of children. Further studies on the incidence of intentional injuries (recreational drug use and suicide intents) in this population, and the effect of medication on the risk of poisoning are needed.

## Methods

The Preferred Reporting Items for Systematic Review and Meta-Analysis Protocols (PRISMA-P)^53,54^, the Preferred Reporting Items for Systematic Review and Meta-Analysis (PRISMA)^55,56^ and the Meta-analysis of Observational Studies in Epidemiology (MOOSE)^57^ guidelines were followed when planning and carrying out our work. The protocol for the study was registered in the international prospective register of systematic reviews held by the University of York (PROSPERO) prior to data analysis (registration number CRD42017079911). Methods reflected those of our previous meta-analysis on the risk of physical injuries in order to make results comparable^58^.

Three major databases –PubMed (Medline Plus), Web of Science core database and Scopus- were searched. Furthermore, we searched over 110 additional databases from an institutional reference aggregator (*UNIKA*: http://www.unav.edu/en/web/biblioteca), that uses the EBSCO discovery service (http://support.ebsco.com/help/index.php?lang=en&int=eds) to provide a list of references combining both internal (library) and external (database vendors) sources. Searches were carried out on November 30^th^ 2017, with no time or language restrictions. References of retrieved pertinent papers were scanned to find additional possibly relevant studies. References of interest from our previous meta-analysis on the risk of physical injuries were also evaluated for potential inclusion^17^. See additional details, including search syntax, in Supplementary Methods 1 and 2 (Supplementary material).

### Study selection

#### Study type

Data from published or unpublished empirical studies that compared the risk of poisoning in children and/or adolescents with ADHD and in typically developing individuals were combined regardless of the design, the temporality (i.e., prospective, retrospective or cross-sectional) or setting (clinical or epidemiological).

ADHD diagnosis is more common in males^59^ and, similarly, poisoning injuries occur more frequently in males than in females^1^. Therefore, we included only articles that took into account this bias either by sample selection (no differences in the number of male and females between the ADHD and no ADHD samples) or statistically (sex controlled as a confounding covariate).

#### Population

The majority of the sample of a study had to be children and/or adolescents (defined as less than 18 years-old). The accepted operational definitions of ADHD were the following: (1) A categorical diagnosis according to standardized criteria, either the DSM (III, III-R, IV, IV-TR or 5) or the diagnosis of hyperkinetic disorder as per ICD-10 or previous versions; (2) Being above a pre-established threshold in a validated psychometric scale for the screening of ADHD; (3) The coding of the diagnosis in a medical registry; (4) A positive answer to the question: “Have you ever been told by a doctor that you have ADHD?” or similar questions; and (5) Being prescribed ADHD medication(s). Studies on preschoolers and those using the diagnosis of “Deficits in attention, motor control, and perception” (DAMP)^60^, or equivalent constructs^61^, were excluded, since they are not equivalent to ADHD.

#### Outcomes

The World Health Organization (WHO) definition of poisoning or intoxication was used to define eligible outcomes. Intoxication is defined by WHO as “a condition that follows the administration of a psychoactive substance and results in disturbances in the level of consciousness, cognition, perception, judgment, affect, or behavior, or other psychophysiological functions and response”1. Since the term “intoxication” can be used in relation to alcohol or drug abuse, outcomes describing poisoning were preferred to those reporting intoxications. However, if an article only reported intoxications, it was also included. Hence, outcomes from articles reporting health problems related to the codes T36-T61 of the 19th chapter of the International Classification of Diseases (ICD-10) or similar problems were deemed eligible.

Poisoning cases had to be attended at medical settings and a registry created or self-reported. Studies reporting information requests to poison information centers or similar entities were not included. Poisoning could occur before or after the diagnosis of ADHD. Risk measures had to describe the ratio in the risk of poisoning between children and adolescents with and without ADHD. The primary outcome measure was defined as the hazard ratio (HR) obtained from Cox proportional hazards models, as it provides a time-independent estimation of the risk. However, odds ratios (ORs) are more frequently reported than HR, and they are the only risk measure that can be obtained from case-control studies that compare ADHD rates in a poisoned and a non-poisoned group. Therefore, ORs were accepted as secondary valid estimations of relative risk and combined with HRs.

In order to address the secondary aim of the present meta-analysis (i.e., assess if there is a significantly higher relative risk of poisoning compared to the relative risk of suffering other physical injuries), studies which reported outcomes on the relative risk of physical injuries in general and also provided similar data on the relative risk of poisoning were eligible.

### Identification and selection of studies

Studies were identified and selected following a two-stage process: (1) Two investigators independently and blindly screened retrieved titles and abstracts of all non-duplicated papers to exclude non-pertinent ones. Discrepancies were resolved by consensus; (2) Articles carried to this stage were assessed after reading the full-text following a similar process of double evaluation similar to the one of the previous step. Multiple reports of the same study were linked together.

### Data extraction

M.R.-G. and G.A. independently extracted data from articles that had been deemed eligible in the selection stage.

A modified version of the Newcastle-Ottawa Scale (NOS)37 was used to assess the risk of bias of each study and rated independently by the same authors. Any discrepancies at this stage were resolved by consensus between M.R.-G. and G.A. Two items, one in the selection and one in the exposure subsections, were eliminated as they were deemed inadequate for our study. Hence, the maximum score in the scale was seven instead of nine stars. Final items of the scale can be found in Supplementary Methods 3 in supplementary material.

### Data synthesis

Summary effect measures (HRs or ORs) were combined in order to estimate a population-average relative risk between ADHD and poisoning in children and adolescents. Hazard Ratios and Odds Ratios were considered equivalent measures of relative risk as the baseline prevalence of poisoning was expected to be very low (less than 1%). In such cases the two measures yield very close results^62,63^.

Robust Variance Estimation^64,65^, a statistical technique that models the nested structure between outcomes of the same study and allows to account for the non-independence of outcomes, was used for the inference of a mean effect size and meta-regression analyses. We carried out a mixed-effect model with robust variance and random-effect estimates. A model with variation of RRs between studies and equicorrelation between same-study effect sizes (p; p = 0.8 in this case) was assumed. This strategy is highly efficient to estimate a mean model from outcomes which are typically correlated at the study level, but are usually independent between studies^64^. The influence of the equicorrelation value chosen here, the most commonly used in previous studies^65,66^, was evaluated in a sensitivity analysis with varying levels of p (0.1 steps between 0 and 1).

Cochran’s Q test and I^2^ index^35^ were used to evaluate heterogeneity among studies, whereas Begg’s adjusted rank correlation and Egger’s test were implemented to formally assess the presence of “small-sample” bias. These analyses were carried out using a single outcome per study. This outcome was selected at random whenever more than one existed. We planned to combine all outcomes fulfilling our inclusion criteria independently of the results of heterogeneity analyses and deal with heterogeneity through the use of a random-effects model and meta-regression, as the exclusion of studies prior to performing a meta-analysis affects the validity of the subsequent results^67^. Additional sensitivity/subgroup analyses consisted in: 1-Including ORs and HRs in separate analyses; 2-Calculating a mean effect size including only statistically unadjusted outcomes (from studies that, at least, controlled for sex by design); 3-Calculating a mean effect size including only adjusted outcomes (controlled covariates could include sex. If this was not the case, sex was controlled through sample selection); 4-Evaluating the influence of removing articles that report intoxication risk (instead of poisoning); 5-Investigating the influence of the risk of bias as evaluated by the rating in the Newcastle-Ottawa Scale by carrying out a meta-regression analysis with the number of stars on the scale for each article as a predictor. We also investigated the effect of age on the risk of poisoning by splitting outcomes into 3 groups according to the age distribution of the participants: outcomes in which participants were under the age of 10, outcomes that included individuals of any age and outcomes in which participants were between 11 and 18 years old. The three groups of outcomes were compared pair-wise using a between-study meta-regression model as we hypothesized that the risk of poisoning would be significantly greater in the older group when compared to younger individuals or to individuals of all ages.

Finally, we investigated whether, compared to those without, children/adolescents with ADHD had a significantly higher risk of poisoning than of suffering other kinds of physical injuries. To this end, in order to control for confounding variables which could affect this comparison (i.e. country of origin and socio-cultural background of participants), only studies that reported both outcomes (effect measure of poisoning and of suffering physical injuries) were evaluated. These two groups of outcomes were compared using a within-study meta-regression model^64,65^. This analysis is optimal in cases where there exists within-studies variability in the covariate (outcome type in our case). This variability is studied by including the distance value around the study regressor mean as a covariate in the regression model^68^.

Effect sizes whose 95% confidence intervals (CIs) did not include 1 were considered significant. Analyses were carried out in STATA v13. Forest plots were created using the DistillerSR Forest Plot Generator from Evidence Partners (https://www.evidencepartners.com/resources/forest-plot-generator/).

### Data statement

All data used in the preparation of the systematic review and meta-analysis is available upon request.

## Electronic supplementary material

Supplementary material
