# Supplementary material for: Risk of poisoning in children and adolescents with ADHD: a systematic review and meta-analysis
Source: Sci Rep. 2018 May 15;8:7584. doi: 10.1038/s41598-018-25893-9 (PMC5953942; doi:10.1038/s41598-018-25893-9)
Supplement: Supplementary file 1 — Supplementary material [file 41598_2018_25893_MOESM1_ESM.pdf]

Supplementary Material
Risk of Poisoning in children and adolescents with ADHD: a systematic Review and Meta-analysis.

Maite Ruiz-Goikoetxea[1]; Samuele Cortese[23456]; Sara Magallón[7]; Maite Aznarez-Sanado[7,] Noelia Alvarez-Zallo[1]; Elkin O. Luis[7]; Pilar de Castro-Manglano[8]; Cesar Soutullo[8]; Gonzalo Arrondo[9]*

*Corresponding author

Correspondence to: garrondo@yahoo.es

1: Servicio de Urgencias Extrahospitalarias Servicio Navarro de Salud-Osasunbidea, Pamplona, Spain.

2: Center for Innovation in Mental Health, University of Southampton, Academic Unit of Psychology, Southampton, UK

3: Faculty of Medicine, Clinical and Experimental Sciences (CNS and Psychiatry),

University of Southampton, Southampton, UK

4: Department of Child and Adolescent Psychiatry, NYU Langone Medical Center, New York, NY, USA

5: Solent NHS Trust, Southampton, UK

6: Division of Psychiatry and Applied Psychology, School of Medicine, University of Nottingham, Nottingham, UK

7: Facultad de Educación y Psicología, Universidad de Navarra, Pamplona, Spain

8: Departamento de Psiquiatría y Psicología Médica, Unidad de Psiquiatría Infantil y Adolescente, Clínica Universidad de Navarra, Pamplona and Madrid, Spain

9: Instituto Cultura y Sociedad (ICS), Grupo Mente-Cerebro, Universidad de Navarra, Pamplona, Spain

Index

Index 2

Methods S1: Search and syntax for each database. 3

Methods S2: Databases included in the UNIKA Service. 4

Methods S3: Risk of bias (Items from the Newcastle-Ottawa Scale). 5

Table S1: Articles excluded with main reason for exclusion. 8

Table S2: Outcomes included in each analysis. 10

Table S3: Description of all outcomes included in the risk of physical injuries *vs.* risk of poisoning analysis. 11

Table S4: PRISMA checklist 13

References 16

Methods S1: Search and syntax for each database.

The following databases were searched

- PubMed (Medline Plus)
- Scopus
- Web of Science Core Collection,
- UNIKA (An institutional reference aggregator that searches in 114 databases listed in the following section)

The following search syntax was used:

PubMed (Medline Plus) and Unika

(In the case of PubMed-Medline Plus, the search was not limited to any field. In the case of Unika, the search was limited to titles, keywords, and abstracts through the website options. The medicine profiles was used for the Unika search)

(ADHD OR adhd OR attention deficit disorder with hyperactivity OR syndrome hyperkinetic OR hyperkinetic syndrome OR hyperactivity disorder OR hyperactive child syndrome OR childhood hyperkinetic syndrome OR attention deficit hyperactivity disorders OR attention deficit hyperactivity disorder OR adhd attention deficit hyperactivity disorder OR adhd OR overactive child syndrome OR attention deficit hyperkinetic disorder OR hyperkinetic disorder OR attention deficit disorder hyperactivity OR attention deficit disorders hyperactivity OR child attention deficit disorder OR hyperkinetic syndromes OR syndromes hyperkinetic OR hyperkinetic syndrome childhood) AND (intox* OR overdos* OR poison*)

Scopus

TITLE-ABS-KEY ((ADHD OR adhd OR “attention deficit disorder with hyperactivity” OR “syndrome hyperkinetic” OR “hyperkinetic syndrome” OR “hyperactivity disorder” OR “hyperactive child syndrome” OR “childhood hyperkinetic syndrome” OR “attention deficit hyperactivity disorders” OR “attention deficit hyperactivity disorder” OR “adhd attention deficit hyperactivity disorder” OR adhd OR “overactive child syndrome” OR “attention deficit hyperkinetic disorder” OR “hyperkinetic disorder” OR “attention deficit disorder hyperactivity” OR “attention deficit disorders hyperactivity” OR “child attention deficit disorder” OR “hyperkinetic syndromes” OR “syndromes hyperkinetic” OR “hyperkinetic syndrome childhood”) AND (intox* OR overdos* OR poison*))

Web of Science:

(TS= ((ADHD OR adhd OR “attention deficit disorder with hyperactivity” OR “syndrome hyperkinetic” OR “hyperkinetic syndrome” OR “hyperactivity disorder” OR “hyperactive child syndrome” OR “childhood hyperkinetic syndrome” OR “attention deficit hyperactivity disorders” OR “attention deficit hyperactivity disorder” OR “adhd

attention deficit hyperactivity disorder" OR adhd OR "overactive child syndrome" OR "attention deficit hyperkinetic disorder" OR "hyperkinetic disorder" OR "attention deficit disorder hyperactivity" OR "attention deficit disorders hyperactivity" OR "child attention deficit disorder" OR "hyperkinetic syndromes" OR "syndromes hyperkinetic" OR "hyperkinetic syndrome childhood") AND (intox* OR overdos* OR poison*)) OR TI= ((ADHD OR adhd OR "attention deficit disorder with hyperactivity" OR "syndrome hyperkinetic" OR "hyperkinetic syndrome" OR "hyperactivity disorder" OR "hyperactive child syndrome" OR "childhood hyperkinetic syndrome" OR "attention deficit hyperactivity disorders" OR "attention deficit hyperactivity disorder" OR "adhd attention deficit hyperactivity disorder" OR adhd OR "overactive child syndrome" OR "attention deficit hyperkinetic disorder" OR "hyperkinetic disorder" OR "attention deficit disorder hyperactivity" OR "attention deficit disorders hyperactivity" OR "child attention deficit disorder" OR "hyperkinetic syndromes" OR "syndromes hyperkinetic" OR "hyperkinetic syndrome childhood") AND (intox* OR overdos* OR poison*)))

Methods S2: Databases included in the UNIKA Service.

A search was carried out in UNIKA (http://www.unav.edu/en/web/biblioteca), an institutional reference aggregator that uses the EBSCO discovery service (http://support.ebsco.com/help/index.php?lang=en&int=eds) to provide a combined list of references from both internal (library) and external (database vendors) sources. The databases included in the biomedical sciences profile of the UNIKA Service from the University of Navarra are listed here in alphabetical order:

1. Academic Search Index (asx)
2. AccessAnesthesiology
3. AccessMedicine
4. AccessPediatrics
5. AccessScience
6. AccessSurgery
7. Ambrose Digital Library
8. ASM Handbooks Online (edsaho)
9. ASM Medical Materials Database
10. ASM Micrograph Database
11. BioOne Online Journals
12. Books at JSTOR
13. British Library Document Supply Centre Inside Serials & Conference Proceedings (edsbl)
14. British Standards Online
15. Business Source Complete
16. Canadian Electronic Library
17. Catálogo de la Biblioteca de la Universidad de Navarra (cat00378a)
18. Center for Research Libraries
19. ChemSpider
20. China/Asia On Demand
21. CINAHL (cin20)
22. CogPrints
23. Credo Reference Collections (edscrc)
24. DADUN (ir00048a)
25. DASH
26. Data-Planet Statistical Datasets & Statistical Ready Reference
27. Dialnet
28. Directory of Open Access Journals (edsdoj)
29. eArticle
30. eBook Academic Collection (EBSCOhost) (e000xww)
31. eBook Collection (EBSCOhost) (nlebk)
32. EconLit (ecn)
33. EDS Foundation Index (eda)
34. eLibro Premium
35. ERIC (eric)
36. eScholarship (edssch)
37. EThOS
38. EU Bookshop (edseub)
39. European Union Open Data Portal
40. Europeana
41. Expanded Academic ASAP
42. Films on Demand
43. Fuente Académica Premier (fua)
44. Gale Cengage Learning, Health & Wellness Resource Center
45. Gale Virtual Reference Library
46. Gallica Bibliothèque Numérique

47. Google Book Search (fe334f7c)
48. GreenFILE (8gh)
49. Harvard Library Bibliographic Dataset (edshlc)
50. HathiTrust (edshtl)
51. Henry Stewart Talks
52. HighWire Press (fa0f9666)
53. Idunn.no
54. IndianJournals.com
55. Informit Health Collection (edsihc)
56. Iprbooks
57. JSTOR (fd43b2a1)
58. JSTOR Life Sciences (edsjls)
59. KERIS Theses & Dissertations (edsker)
60. Knigafund.ru (edskig)
61. Korean Studies Information Service System (KISS) (edskis)
62. LexisNexis Academic: Law Reviews (edslex)
63. Maruzen eBook Library
64. McGraw-Hill
65. Medical Online
66. Medical Online E-books
67. Medical Online-E
68. MEDLINE (cmedm)
69. Minority Health Archive (edsuph)
70. NARCIS
71. Networked Digital Library of Theses & Dissertations (edsndl)
72. NORA (Norwegian Open Research Archive)
73. OAIster (edsoai)
74. OJS vid Lunds Universitet (edsojs)
75. Ovid Journals Full Text Medical Research Database (fb0698e8)
76. Oxford Bibliographies Online
77. Oxford Clinical Psychology
78. Oxford Handbooks Online (edsoho)
79. Oxford Medicine Online
80. Oxford Reference (edsoro)
81. Oxford Scholarship Online (edsoso)
82. ProQuest Dissertations and Theses (fb458d87)
83. PsycARTICLES (edspdh)
84. PsycBOOKS (edspzh)
85. PsycCRITIQUES (edspvh)
86. PsycheVisual
87. Psychology and Behavioral Sciences Collection (pbh)
88. PsycINFO (psyh)
89. Publisher Provided Full Text Searching File (edb)
90. PubMed Central (fd5a6824)
91. R2 Digital Library
92. RACO
93. RECERCAT
94. ReferenceSearch (edsref)
95. RÖMPP Online
96. SA ePublications Service
97. SAGE Research Methods Datasets
98. SAGE Video
99. Scielo
100. Scielo Books
101. Science Citation Index (edswsc)
102. ScienceDirect (edselp)
103. Scopus
104. Social Sciences Citation Index (edswss)
105. Springer Science+Business Media, SpringerProtocols
106. STAT!Ref
107. Supplemental Index (edo)
108. SveMed+ (edssmd)
109. Torrossa
110. TOXNET: GENETOX
111. TOXNET: TOXLINE
112. University Library Online - Университетская библиотека онлайн
113. World Bank eLibrary (edswb

Methods S3: Risk of bias (Items from the Newcastle-Ottawa Scale).

Studies comparing poisoned vs non-poisoned (case-control studies)
Selection

1) Is the case definition adequate?
a) yes, with independent validation * (acute poisoning or record linkage+interview or other validation)
b) yes, eg. record linkage or based on self-reports
c) no description

2) Representativeness of the cases
a) consecutive or obviously representative series of cases*
b) potential for selection biases or not stated

3) Selection of Controls

a) community controls *
b) hospital controls
c) no description

4) Definition of Controls : IRRELEVANT IN OUR CASE
a) no history of disease (endpoint) *
b) no description of source

Comparability (up to 2 stars)

1) Comparability of poisoned and non-poisoned individuals on the basis of the design or analysis (Note: all articles should control sex for inclusion).
a) study controls for AGE and COMORBIDITY **
b) study controls for AGE *
c) study controls for COMORBIDITY *

Exposure (ADHD)

1) Ascertainment of ADHD
a) secure record (eg surgical records) or data linkage *
b) structured interview *
c) written self-report, (not codified) medical history or clinical questionnaire
d) no description

2) Same method of ascertainment for cases and controls
a) yes *
b) no

3) Non-Response rate: IRRELEVANT IN OUR CASE
a) same rate for both groups *
b) non respondents described
c) rate different and no designation

Cohort studies

Selection

1) Representativeness of the exposed cohort. Individuals with ADHD are
a) truly representative of the average child with ADHD in the community *
b) somewhat representative of the average child with ADHD in the community (individuals may differ slightly from the typical ADHD child)*
c) selected group of users eg only medicated ADHD, all ADHD+Comorbidity, only one sex, only hospitally-treated ADHD.
d) no description of the derivation of the cohort

2) Selection of the non-exposed cohort (individuals without ADHD)
a) drawn from the same community as the exposed cohort *
b) drawn from a different source
c) no description of the derivation of the non-exposed cohort

3) Ascertainment of ADHD
a) secure record (eg surgical records) or data linkage *

b) structured interview *
c) written self-report, (not codified) medical history or clinical questionnaire
d) no description

4) Demonstration that outcome of interest was not present at start of study: IRRELEVANT IN OUR CASE THAT ADHD IS PRESENT BEFORE STUDY STARTS
a) yes *
b) no

Comparability (up to 2 stars)

1) Comparability of individuals with ADHD and no ADHD on the basis of the design or analysis (NOTE: all studies should control for sex)
a) study controls for AGE and comorbidity **
b) study controls for AGE *
c) study controls for comorbidity *

Outcome

1) Assessment of the poisonings
a) independent blind assessment *
b) record linkage *
c) self-report
d) no description

2) Was follow-up long enough for outcomes to occur : IRRELEVANT IN OUR CASE: ANY FOLLOW-UP WAS CONSIDERED ADEQUATE.
a) yes (select an adequate follow up period for outcome of interest) *
b) no

3) Adequacy of follow up of cohorts
a) complete follow up - all subjects accounted for *
b) subjects lost to follow up unlikely to introduce bias - small number lost - >80 % follow up, or description provided of those lost) *
c) follow up rate < 80% and no description of those lost
d) no statement

Table S1: Articles excluded with main reason for exclusion.

Reference	Reason
Ayaz AB, Ayaz M, Şentürk E, Soylu N, Yüksel S, Yulaf Y. Factors related with unintentional injuries in children with newly diagnosed attention-deficit/ hyperactivity disorder. *Int J Inj Contr Saf Promot*. 2016;23(1):93-98.	No control group
Ballinger L. Rave On. CounterPunch. 2013;20(4):14.	Not empirical
Barkla XM, Mcardle PA, Newbury-birch D. Are there any potentially dangerous pharmacological effects of combining ADHD medication with alcohol and drugs of abuse ? A systematic review of the literature. *BMC Psychiatry*. 2015;15(1):15-19.	Not empirical
Barkley RA. Accidents and attention-deficit/hyperactivity disorder. Econ Neurosci. 2001;3(4):64-68.	Not empirical
Barkley RA. ADHD and Accident Proneness. (Cover story). ADHD Rep. 2002;10(2):2-6.	Not empirical
Basavaraj DS, Forster DP. Accidental poisoning in young children. J Epidemiol Community Health. 1982;36(1):31-34.	Pre-school
Bekdas M, Goksugur SB, Balaban A DF. Methylphenidate Poisoning: Carpopedal Spasm in a Child. J Med Cases VO - 5. 2014;(1):31-33.	Not empirical
Cakmak M, Gollu G, Boybeyi O, et al. Cognitive and behavioral characteristics of children with caustic ingestion. J Pediatr Surg. 2015;50(4):540-542.	Scales without threshold
De Alwis D, Agrawal A, Reiersen AM, et al. ADHD symptoms, autistic traits, and substance use and misuse in adult Australian twins. J Stud Alcohol Drugs. 2014;75(2):211-221.	Adults
Gordon S. Abuse of ADHD Drugs on the Rise; Jump in poison control center calls mirrors increasing prescriptions, study shows. Consumer Health News (English).	Not empirical
Harris M. Self-Induced Overdose of Adhd Medication. J Paediatr Child Health. 1997;33(2):176-176.	Not empirical
Hartsough CS, Lambert NM. Pattern and progression of drug use among hyperactives and controls: a prospective short-term longitudinal study. J Child Psychol Psychiatry. 1987;28(4):543-553.	It does not relate ADHD to poisoning
Hawton K, Saunders K, Topiwala A, Haw C. Psychiatric disorders in patients presenting to hospital following self-harm: A systematic review. J Affect Disord. 2013;151(3):821-830.	Systematic Review. No control group
Huang Y-S, Tsai M-H. Long-term outcomes with medications for attention-deficit hyperactivity disorder: current status of knowledge. CNS Drugs. 2011;25(7):539-554..	Not empirical
Katrivanou A, Lekka NP, Beratis S. Psychopathology and behavioural trends of children with accidental poisoning. J Psychosom Res. 2004;57(1):95-101.	Pre-school
Knouse L. Cost of Accidents. ADHD Rep. 2005;13(3):12.	Not empirical
Levine M, Froberg B, Ruha AM, et al. Assessing the toxicity and associated costs among pediatric patients admitted with unintentional poisonings of attention-deficit/hyperactivity disorder drugs in the United States. Clin Toxicol. 2013;51(3):147-150.	No control group
LoVecchio F, Ozimek J, Sawyers B, Thole D. Outcomes after accidental pediatric ingestions of (dextro)amphetamine and methylphenidate. Am J Emerg Med. 2009;27(8):933-934.	It does not relate ADHD to poisoning
McCarthy S, Cranswick N, Potts L, Taylor E, Wong ICK. Mortality associated with attention-deficit hyperactivity disorder (ADHD) drug treatment: A retrospective cohort study of children, adolescents and young adults using the general practice research database. Drug Saf. 2009;32(11):1089-1096.	It does not relate ADHD to poisoning
Minde K. The use of psychotropic medication in preschoolers: some recent	Not empirical

Reference	Reason
developments. Can J Psychiatry. 1998;43(6):571-575.	
Nelson J. Reduce unintentional poisoning. Breckenridge Am. June 2008:5A.	It does not relate ADHD to poisoning
Niemelä S, Sourander A, Poikolainen K, et al. Childhood predictors of drunkenness in late adolescence among males: A 10-year population-based follow-up study. Addiction. 2006;101(4):512-521.	It does not relate ADHD to poisoning
Ramon F, Ballesteros S. Exposures to drugs used to treat attention deficit hyperactivity disorder (ADHD): A Poison Control Center experience. Clin Toxicol. 2016;Conference:36th International Congress of the European Associ.	No control group
Rey JM, Walter G, Hazell PL. Psychotropic drugs and preschoolers. Med J Aust. 2000;173(4):172-173.	Not empirical
Setlik J, Bond GR, Ho M. Adolescent Prescription ADHD Medication Abuse Is Rising Along With Prescriptions for These Medications. Pediatrics. 2009;124(3):875-880.	No control group
Sheikh S, Hendry P, Lynch S, Kalynych CJ, Aldridge P, Kraemer D. Poisonings with Suicidal Intent Aged 0-21 Years Reported to Poison Centers 2003-12. West J Emerg Med. 2015;16(4):497-502.	No control group
Sibert JR, Newcombe RG. Accidental ingestion of poisons and child personality. Postgrad Med J. 1977;53(619):254-256.	It does not relate ADHD to poisoning
Stewart MA, Thach BT, Freidin MR. Accidental poisoning and the hyperactive child syndrome. Dis Nerv Syst. 1970;31(6):403-407.	Sex is uncontrolled
Stoltzfoos L. Abuse of Stimulant Medications By Youths Increases Along With Access. Prim Psychiatry. 2009;16(10):12-13	Not empirical
van den Ban E, Souverein P, Meijer W, et al. Association between ADHD drug use and injuries among children and adolescents. Eur Child Adolesc Psychiatry. 2014;23(2):95-102.	It does not relate ADHD to poisoning
Yule A, Wilens T, Carrellas N. Attention Deficit Hyperactivity Disorder (ADHD) and Overdose (OD) Risk in Transitional Age Youth (TAY) with Substance Use Disorders (SUD). Am J Addict. 2016;25(4):341.	Sex is uncontrolled. It assessed drug overdose on a population with substance use disorder

The reference of the article and a main reason for exclusion from the meta-analysis are provided

Table S2: Outcomes included in each analysis.

	Relative Risk	Main RR of poisoning	HR outcomes	OR outcomes	Adjusted outcomes	Unadjusted outcomes	NOS meta-regression	Under 10 vs. Age unspecified	Over 10 vs. Age unspecified	Under 10 vs. Over 10	Poisoning vs physical injuries
Brehaut (2003)	2.67	1	x	1	1	x	1	1	1	x	1
CPRD-HES: Prasad (2016)	3.99	1	1	x	1	x	1	1	1	x	1
Hariharan (2008)	7.98	1	x	1	x	1	1	0	x	0	x
Hurtig (2016)	1.51	1	1	x	1	x	1	0	x	0	1
Hurtig (2016)	3.42	1	1	x	1	x	1	x	0	1	1
Hurtig (2016)	6.29	1	1	x	1	x	1	x	0	1	1
LHID: Tai (2013)	1.23	x	x	1	x	x	x	x	0	1	x
LHID: Chou (2014)	4.51	x	1	x	x	1	x	x	x	x	x
LHID: Chou (2014)	4.65	1	1	x	1	x	1	x	x	x	1
LHID: Chou (2014)	4.50	x	x	x	x	1	x	x	x	x	x
LHID: Chou (2014)	2.42	x	x	x	x	1	x	0	x	0	x
LHID: Chou (2014)	17.86	x	x	x	x	1	x	x	0	1	x
Lindemann (2017)	3.47	1	1	x	1	x	1	1	1	x	1
Rowe (2004)	1.2	1	x	1	1	x	1	1	1	x	1
Silva (2014)	2.24	1	x	1	1	x	1	0	x	0	1
Swensen (2004)	4.46	1	x	1	x	1	1	1	1	x	1

Table indicates which poisoning outcomes were included in each analysis. X indicates that the outcome was not included in each analysis. 1 indicates that the outcome was included in a meta-analysis or meta-regression analysis and compared to outcomes marked with 0 when appropriate.

Table S3: Description of all outcomes included in the risk of physical injuries *vs.* risk of poisoning analysis.

First author (year)	Measure	Type of injury	Description of outcome	N ADHD	N non-ADHD	Number of non-ADHD injured	Number of ADHD injured	Effect measure	LBCI	UBCI
Brehaut (2003)[1]	OR	Any injury	Adjusted	16806	1010067	32242	1257	1.67	1.57	1.68
CPRD-HES: Hire (2016)[11a]	HR	Fractures	Adjusted	5111	49489	8461	470	1.17	1.06	1.3
CPRD-HES: Prasad (2016)[2]	HR	Fractures	Adjusted	15737	291894	18598	1878	1.28	1.22	1.35
CPRD-HES : Prasad (2016)[3]	HR	Burns	Adjusted	15741	291909	11958	1189	1.23	1.16	1.31
Hurtig (2016)[4]	HR	Any Injury	Rating Scale, injury between 0 and 6 years. Adjusted	875	5236	221	62	1.41	1.03	1.93
Hurtig (2016)[4]	HR	Any Injury	Rating scale, injury between 7 and 15 years. Adjusted	472	5639	383	54	1.45	1.07	1.97
Hurtig (2016)[4]	HR	Any Injury	ADHD diagnosis, injury between 7 and 15 years. Adjusted	105	352	28	15	2.33	1.2	4.51
LHID: Chou (2014)[12a]	HR	Fractures	Adjusted	3640	14560	1188	389	1.26	1.12	1.42
LHID: Kang (2013)[13a]	HR	Any Injury	Adjusted	3616	18080	2908	864	1.64	1.5	1.79
LHID: Guo (2015)[14]	HR	Fractures	Adjusted	7200	36000	2333	645	1.41	1.29	1.54
LHID: Tai (2013)[5]	HR	Any injury	Adjusted	1965	7860	6052	1856	1.7	1.55	2.06
Lindemann (2017)[7]	HR	Any injury	Adjusted	37650	37650	NR	NR	1.4	1.3	1.49

First author (year)	Measure	Type of injury	Description of outcome	N ADHD	N non-ADHD	Number of non-ADHD injured	Number of ADHD injured	Effect measure	LBCI	UBCI
Rowe (2004)[8]	OR	Burns	Psychiatric model. Adjusted	NR	NR	NR	NR	1.5	0.7	2.9
Rowe (2004)[8]	OR	Fractures	Psychiatric model. Adjusted	NR	NR	NR	NR	1.7	1.2	2.4
Silva (2014)[9]	OR	Any Injury	Adjusted	11902	27304	1479	1112	1.73	1.59	1.88
Swensen (2004) [10]	OR	Any Injury	Unadjusted	1308	1308	264	415	1.84	1.54	2.20

Outcome-level details of all the outcomes reporting risk of unintentional physical injuries included in the risk of poisoning vs. risk of UPI; N: number of individuals in each group; OR: odds ratio between ADHD and non-ADHD; HR: hazard ratio between ADHD and non-ADHD; LBCI: lower bound of the 95% confident interval; UBCI: upper bound of the 95% confident interval.. NR: not reported.

a= Articles that do not report poisoning but report unintentional injuries from the same samples (and hence have been considered the same study).

Table S4: PRISMA checklist

Section/topic	*#*	*Checklist item*	*Reported on page #*
**TITLE**			
Title	1	Identify the report as a systematic review, meta-analysis, or both.	1
**ABSTRACT**			
Structured summary	2	Provide a structured summary including, as applicable: background; objectives; data sources; study eligibility criteria, participants, and interventions; study appraisal and synthesis methods; results; limitations; conclusions and implications of key findings; systematic review registration number.	3
**INTRODUCTION**			
Rationale	3	Describe the rationale for the review in the context of what is already known.	4-5
Objectives	4	Provide an explicit statement of questions being addressed with reference to participants, interventions, comparisons, outcomes, and study design (PICOS).	5-6
**METHODS**			
Protocol and registration	5	Indicate if a review protocol exists, if and where it can be accessed (e.g., Web address), and, if available, provide registration information including registration number.	17
Eligibility criteria	6	Specify study characteristics (e.g., PICOS, length of follow-up) and report characteristics (e.g., years considered, language, publication status) used as criteria for eligibility, giving rationale.	18-19
Information sources	7	Describe all information sources (e.g., databases with dates of coverage, contact with study authors to identify additional studies) in the search and date last searched.	17. S4-S5
Search	8	Present full electronic search strategy for at least one database, including any limits used, such that it could be repeated.	S3-S4

Section/topic	#	*Checklist item*	*Reported on page #*
Study selection	9	State the process for selecting studies (i.e., screening, eligibility, included in systematic review, and, if applicable, included in the meta-analysis).	18-19
Data collection process	10	Describe method of data extraction from reports (e.g., piloted forms, independently, in duplicate) and any processes for obtaining and confirming data from investigators.	20-21
Data items	11	List and define all variables for which data were sought (e.g., PICOS, funding sources) and any assumptions and simplifications made.	In protocol
Risk of bias in individual studies	12	Describe methods used for assessing risk of bias of individual studies (including specification of whether this was done at the study or outcome level), and how this information is to be used in any data synthesis.	22
Summary measures	13	State the principal summary measures (e.g., risk ratio, difference in means).	19
Synthesis of results	14	Describe the methods of handling data and combining results of studies, if done, including measures of consistency (e.g., I^2) for each meta-analysis.	21-22
Risk of bias across studies	15	Specify any assessment of risk of bias that may affect the cumulative evidence (e.g., publication bias, selective reporting within studies).	21-22
Additional analyses	16	Describe methods of additional analyses (e.g., sensitivity or subgroup analyses, meta-regression), if done, indicating which were pre-specified.	21-22
RESULTS			
Study selection	17	Give numbers of studies screened, assessed for eligibility, and included in the review, with reasons for exclusions at each stage, ideally with a flow diagram.	6, Figure 1
Study characteristics	18	For each study, present characteristics for which data were extracted (e.g., study size, PICOS, follow-up period) and provide the citations.	6-8, tables 2 and 4
Risk of bias within studies	19	Present data on risk of bias of each study and, if available, any outcome-level assessment (see Item 12).	8, table 3

Section/topic	#	*Checklist item*	*Reported on page #*
Results of individual studies	20	For all outcomes considered (benefits or harms), present, for each study: (a) simple summary data for each intervention group and (b) effect estimates and confidence intervals, ideally with a forest plot.	Figures 2 and 4, table 4
Synthesis of results	21	Present results of each meta-analysis done, including confidence intervals and measures of consistency.	8-10, Figures 2 and 4, Table S2
Risk of bias across studies	22	Present results of any assessment of risk of bias across studies (see Item 15).	7, Figure 3.
Additional analysis	23	Give results of additional analyses, if done (e.g., sensitivity or subgroup analyses, meta-regression [see Item 16]).	8-10
DISCUSSION			
Summary of evidence	24	Summarize the main findings including the strength of evidence for each main outcome; consider their relevance to key groups (e.g., health care providers, users, and policy makers).	10-17
Limitations	25	Discuss limitations at study and outcome level (e.g., risk of bias), and at review level (e.g., incomplete retrieval of identified research, reporting bias).	10-17
Conclusions	26	Provide a general interpretation of the results in the context of other evidence, and implications for future research.	10-17
FUNDING			
Funding	27	Describe sources of funding for the systematic review and other support (e.g., supply of data); role of funders for the systematic review.	30

PRISMA checklist indicating the location within the article of the recommended content. Pages correspond to the unformatted manuscript for reviewers. The PRISMA checklist is distributed under the terms of the Creative Commons Attribution License, which permits unrestricted use, distribution, and reproduction in any medium, provided the original author and source are credited. The original publication is: Liberati, A. *et al.* The PRISMA statement for reporting systematic reviews and meta-analyses of studies that evaluate healthcare interventions: explanation and elaboration. *BMJ* 339, b2700 (2009).

References

1. Brehaut, J. C., Miller, A., Raina, P. & McGrail, K. M. Childhood behavior disorders and injuries among children and youth: a population-based study. *Pediatrics* **111,** 262–9 (2003).

2. Prasad, V. The epidemiology of injuries in epilepsy and attention deficit-hyperactivity disorder (ADHD) in children and young people using the Clinical Practice Research Datalink (CPRD) and linked data. (University of Nottingham, 2016).

3. Hariharan, S. & Pomerantz, W. Correlation between hospitalization for pharmaceutical ingestion and attention deficit disorder in children aged 5 to 9 years old. *Clin. Pediatr. (Phila).* **47,** 15–20 (2008).

4. Hurtig, T., Ebeling, H., Jokelainen, J., Koivumaa-Honkanen, H. & Taanila, A. The Association Between Hospital-Treated Injuries and ADHD Symptoms in Childhood and Adolescence: A Follow-Up Study in the Northern Finland Birth Cohort 1986. *J. Atten. Disord.* **20,** 3–10 (2016).

5. Tai, Y.-M., Gau, S. S.-F. & Gau, C.-S. Injury-proneness of youth with attention-deficit hyperactivity disorder: a national clinical data analysis in Taiwan. *Res. Dev. Disabil.* **34,** 1100–8 (2013).

6. Chou, I.-C. C. I.-C. *et al.* Attention-deficit hyperactivity disorder increases the risk of deliberate self-poisoning: A population-based cohort. *Eur. Psychiatry* **29,** 523–527 (2014).

7. Lindemann, C., Langner, I., Banaschewski, T., Garbe, E. & Mikolajczyk, R. T. The Risk of Hospitalizations with Injury Diagnoses in a Matched Cohort of Children and Adolescents with and without Attention Deficit/Hyperactivity Disorder in Germany: A Database Study. *Front. Pediatr.* **5,** (2017).

8. Rowe, R., Maughan, B. & Goodman, R. Childhood Psychiatric Disorder and Unintentional Injury: Findings from a National Cohort Study. *J. Pediatr. Psychol.* **29,** 119–130 (2004).

9. Silva, D. *et al.* Children diagnosed with attention deficit disorder and their hospitalisations: population data linkage study. *Eur. Child Adolesc. Psychiatry* **23,** 1043–1050 (2014).

10. Swensen, A. *et al.* Incidence and costs of accidents among attention-deficit/hyperactivity disorder patients. *J. Adolesc. Health* **35,** 346.e1-9 (2004).

11. Hire, A. J. ADHD incidence, treatment and associated comorbidity in children and adolescents : an epidemiological study using electronic healthcare records. (University of Manchester, 2016).

12. Chou, I.-C., Lin, C.-C., Sung, F.-C. & Kao, C.-H. Attention-deficit-hyperactivity disorder increases risk of bone fracture: a population-based cohort study. *Dev. Med. Child Neurol.* **56,** 1111–1116 (2014).

13. Kang, J.-H., Lin, H.-C. & Chung, S.-D. Attention-deficit/hyperactivity disorder increased the risk of injury: a population-based follow-up study. *Acta Paediatr.* **102,** 640–643 (2013).

14. Guo, N.-W. *et al.* Fracture risk and correlating factors of a pediatric population with attention deficit hyperactivity disorder: a nationwide matched study. *J. Pediatr. Orthop. B* **25,** 369–74 (2016).
